# Avian Influenza A Virus Associations in Wild, Terrestrial Mammals: A Review of Potential Synanthropic Vectors to Poultry Facilities

**DOI:** 10.3390/v12121352

**Published:** 2020-11-26

**Authors:** J. Jeffrey Root, Susan A. Shriner

**Affiliations:** U.S. Department of Agriculture, Animal and Plant Health Inspection Service, Wildlife Services, National Wildlife Research Center, Fort Collins, CO 80521, USA; susan.a.shriner@usda.gov

**Keywords:** avian influenza A virus, mammals, peridomestic, review, synanthropic, synthesis, wildlife

## Abstract

The potential role of wild mammals in the epidemiology of influenza A viruses (IAVs) at the farm-side level has gained increasing consideration over the past two decades. In some instances, select mammals may be more likely to visit riparian areas (both close and distant to farms) as well as poultry farms, as compared to traditional reservoir hosts, such as waterfowl. Of significance, many mammalian species can successfully replicate and shed multiple avian IAVs to high titers without prior virus adaptation and often can shed virus in greater quantities than synanthropic avian species. Within this review, we summarize and discuss the potential risks that synanthropic mammals could pose by trafficking IAVs to poultry operations based on current and historic literature.

## 1. Introduction

During recent years, avian influenza A viruses (IAVs) have had devastating consequences to the poultry industry in multiple countries. For example, highly pathogenic H5 IAVs were responsible for death or culling tens of millions of birds in the U.S. during 2015 and the initial costs associated with these outbreaks were estimated to be in the realm of billions of U.S. dollars [1]. Similarly, the early poultry industry cost estimates following the emergence of an H7N9 IAV in China were thought to be greater than 1 billion U.S. dollars [2]. Because of this, it is important to address all facets of the epidemiology of these viruses, including animal species not customarily thought to be involved in the transmission and/or trafficking of IAVs.

While wild birds, such as waterfowl and shorebirds, have long been considered the primary hosts of avian IAVs [3], several recent accounts of natural or experimental susceptibility to multiple avian viruses have been recently described in a diversity of mammal species [4,5,6,7]. As such, some papers have suggested mammals as a potential biosecurity threat to poultry operations [8,9]. A mammalian synanthrope can be defined as a species that lives near or benefits from environments modified by humans [10]. Therefore, mammals that exhibit these behaviors are the most likely species to impose the biosecurity threats mentioned above. For example, in some instances, small mammals may have the capacity to readily enter poultry barns [11], while this is unlikely to be possible for the traditional avian reservoirs mentioned above.

Although reviews have been conducted on some aspects of IAVs in mammals primarily during earlier time periods [12,13,14,15], a large portion of the experimental infection studies in mammalian wildlife, as well as the documentation of several new mammalian species naturally exposed to various IAVs, have been conducted and/or reported in the most recent decade and few of the pervious reviews have focused on terrestrial, synanthropic wildlife. Furthermore, some of the most significant IAV outbreaks in poultry in North America have occurred during the past several years. Because of this, the results of the wildlife epidemiological investigations associated with the U.S. outbreak are discussed.

**Figure 1:**
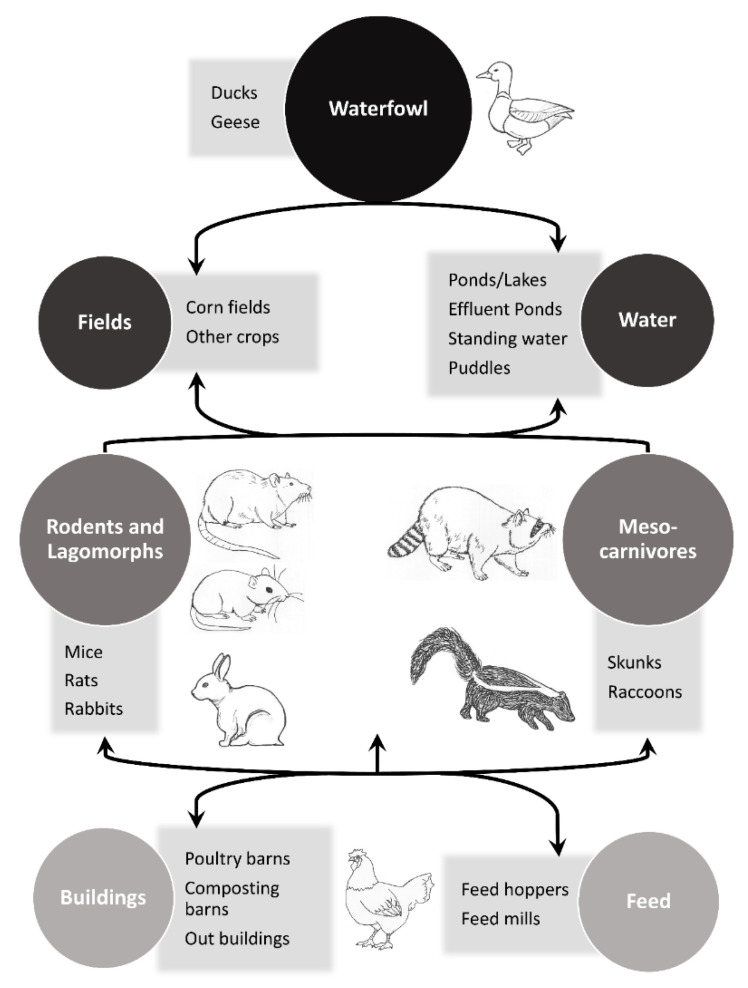
Conceptual model of potential steps for mammals to traffic an avian influenza A virus to or near a poultry facility in North America. Various species, such as Old World mesocarnivores, could be substituted in this diagram to make it applicable to other regions. Figure modified from [8].

The objective of this paper is to review the literature of key non-volant mammalian synanthropes that have evidence of natural exposures to IAVs or have successfully replicated avian IAVs in experimental settings and relate this information to poultry biosecurity. The focus is on wild mammalian associations with avian IAVs; however, instances where avian-origin IAVs are isolated from other species (i.e., avian-origin H7N9 isolated from a human), instances where the origin of the IAV is unknown, and select captive wildlife (e.g., farmed animals and animals in enclosures for conservation purposes) are included. In addition, select examples of mammals that are unlikely to be synanthropic, but other members within their taxonomic families are likely to possess this trait, are included in this review to bring attention to IAV exposures in these groups of mammals. The concept of potential mammalian involvement in trafficking IAVs to and/or near poultry facilities is outlined in a conceptual model (Figure 1). In general, mammals that directly or indirectly interact with waterfowl and are attracted to poultry production facilities are species of potential concern for the movement of IAVs to these types of facilities. In addition, small mammals that can readily enter and move between poultry barns are also of interest.

## 2. Antibody Detections

Antibodies to IAVs have been detected in a diversity of mammals (Table 1). Examples have ranged from small rodents to large carnivores and perissodactlys [16,17]. While the following text focuses on mammals that are known to be synanthropes or could exhibit these tendencies in certain situations, many other species that do not typically exhibit these behavioral traits have produced evidence of antibodies to various IAVs. For example, a recent paper provided evidence of IAV exposure in at least eight species of African mammals, most of which would typically not be considered as synanthropes, with a higher seroprevalence and greater subtype diversity in carnivorous species that commonly feed upon bird species [17]. Because these species are unlikely candidates to exhibit synanthropic tendencies, they are less likely to have close contact with humans or poultry; therefore, the less common interactions of these species at the farm level suggest that their role in trafficking avian IAVs to poultry operations is probably insignificant in most instances [18].

### 2.1. Lagomorphs

Influenza A virus antibody detections in plateau pika (aka black-lipped pika; *Ochotona curzoniae*) have become commonplace at study areas near Qinghai Lake, China. For example, 11 of 82 (13.4%) pika sera tested were shown to be positive for antibodies reactive with H5 subtypes [19]. More recently, 92 of 293 (31.4%) of pika sera tested yielded detectable HI antibody titers against an H9N2 IAV [7]. Antibody detections within the genus *Ochotona* are not limited to plateau pika, as one of 16 Daurian pika (*O. dauurica*) was assessed to be IAV antibody positive in southern Russia near its border with China and Mongolia [16].

### 2.2. Procyonids

Exposure of raccoons (*Procyon lotor*) to IAVs has been assessed in multiple states in the U.S. However, antibody prevalence estimates have ranged widely from 0% in select states (e.g., Georgia, California, Texas, and Louisiana) to moderate/relatively high prevalences in Colorado (12.8%) and Wyoming (25%) [22]. This initial effort has sparked additional antibody investigations in feral/invasive populations of this species. For example, antibodies reactive with multiple H5N1 viruses of different clades were detected in Japan [23]. Similar to results found in the U.S. [22], a second serosurvey in Japan found that IAV antibody detections in raccoons were patchy, with all of the antibody detections occurring among 3 of 18 of the locations surveyed [24].

### 2.3. Canids

Exposure of wild canids to IAVs have been uncommonly reported, likely due to the fact that few surveys have targeted them. Nonetheless, a low (0.4%) prevalence of IAV antibodies were recently reported in Arctic foxes (*Vulpes lagopus*) in the Arctic Coastal Plain of northern Alaska [25]. The same serologic survey, however, did not detect IAV antibodies among 100 polar bear (*Ursus maritimus*) serum samples tested from the same region [25]. The black-backed jackal (*Canis mesomelas*) can be found in variable habitats, including human habitations, suburbs, and deserts [32], thereby suggesting it is synanthropic in some locations. Recently, four of ten black-backed jackals, a species whose diet commonly includes birds, were reported to have antibodies reactive with H3 and H5 antigens in Namibia [17]. In addition, canids unlikely to exhibit synanthropic behaviors have shown evidence of IAV exposure. For example, antibodies reactive with H9 and H7 IAVs were recently reported in wolves (*Canis lupus*; subspecies and if animals were wild or “semi-wild” was not listed in reference) [33]. Furthermore, a serosurvey of farmed raccoon dogs (*Nyctereutes procyonoides*) reported an IAV seropositive rate of 41.4% [26]. These animals were fed uncooked poultry products and were raised on a farm with mink and foxes [26].

### 2.4. Felids

While most species are typically not found in synanthropic settings, several wild felids have shown evidence of exposure to IAVs on multiple continents [16,17,27]. In Russia, for example, multiple cat species were shown to have IAV antibodies [27]. Of the felid species tested in that study which could potentially exhibit synanthropic behavior, one of 15 far-eastern wild cats (aka Amur leopard cat; *Prionailurus bengalensis euptilurus*) were assessed to be seropositive for IAVs [27]. In addition, a single leopard cat (*Prionailurus bengalensis*) was assessed to be antibody positive for IAV in Korea, likely exposed to an H5 IAV as determined by HI tests [28]. Of interest, leopard cat habitat is quite variable, but can include plantation forests and farmland [34]. Furthermore, this species is known to predate upon poultry in some parts of its range [35]. Taken together, these attributes suggest that this species is synanthropic in some settings. Although it is very unlikely to exhibit synanthropic behavior due to the remote habitats in which it subsists, over twenty-two percent of Pallas’s cat (*Otocolobus manul*) serum samples collected in Russia produced IAV seropositive reactions [16]. This species was assumed to have been exposed to IAVs during the consumption of various prey species such as birds, rodents and lagomorphs and/or through contact with bird excrement associated with water-marsh complexes [16]. Similar to Pallas’s cat, caracals (*Caracal caracal*) are likely rarely found in synanthropic settings. Nonetheless, four of eight caracals, a felid species that commonly eats birds, sampled in Namibia had antibodies against a total of 10 hemagglutinins combined [17]. In addition, a small percentage of feral cats were IAV antibody positive in Florida, U.S. by ELISA, but could not be confirmed by HI assays [36]. Considering multiple IAVs (e.g., H5N1 and H7N2) have been detected in domestic cats [29,37], wild and feral felids may warrant surveillance efforts for various IAVs.

### 2.5. Mustelids

Results of IAV surveillance in an invasive population of American mink (*Neovison vison*) were recently reported from Europe. Although 15 of 689 (2.2%) of mink tested by ELISA showed evidence of antibodies reactive to IAVs in the generic sense, none were confirmed by HI assays as being positive for relevant swine (H1N1 and H3N2) or avian (H5N1, H5N3, H7N1, H7N7, and H10N4) IAVs [38]. A second IAV serological survey was conducted in free-ranging American mink in Argentina. This study did not detect IAV antibodies in any of the mink sampled [39]. Of interest, low levels of antibodies were observed in this species following experimental infections with avian-derived viruses; this was in contrast to mink inoculated with mammalian-derived strains, which tended to yield high antibody titers [40]. Nonetheless, high antibody prevalence rates have been reported primarily with H9N2 viruses in farmed mink fed raw poultry and poultry byproducts [41]. Whether mink are farmed or endemic, the occasional escapees of farmed mink can lead to feral populations of this species in multiple regions [42,43]. Overall, a wide variety of mustelids and allies have shown evidence of infection with various IAVs [5].

### 2.6. Rodents

Several IAV antibody detections in wild rodents and other small mammal species have been reported. Various rodent species, especially those that are considered commensal, are known to be commonly found on poultry farms, often while seeking out poultry feed [44]. Hemagglutination inhibiting activity was detected in rat sera from unidentified “rats” associated with a wholesale poultry market in Hong Kong [20], as well as from “sewage rats” (presumably *Rattus* sp.) from select locations in Egypt [21]. In the latter study, two of 72 rats were reported to have antibodies reactive with H5N1 as determined by HI assays [21]. Of the 14 rodents tested from a gamebird farm in Idaho, 6 house mice were presumptively positive for antibodies reactive with IAV using an indirect ELISA [4]. In a serosurvey for pathogens in Pallas’s cat prey items, IAV antibodies were reported from 73.1% of 52 hamsters (*Cricetulus pseudogriseus* and *Phodopus campbelli* combined), 57.7% of 26 Brandt’s voles (*Lasiopodomys brandtii*), 73.7% of 19 Mongolian gerbils (*Meriones unguiculatus*), and 91.3% of 23 Daurian sousliks (*Spermophilus dauricus*) in an arid region of southern Russia [16]. The authors attributed these exposures to the rodents living adjacent to large numbers of wild bids associated with water marsh complexes, and possibly through interactions with bird excrement and bird carcasses for some species [16].

### 2.7. Artiodactyls

Influenza A virus antibodies were recently reported from what might be considered an unlikely group of mammals, the artiodactyls. For example, antibodies to IAVs, as assessed by ELISA and confirmed as H5 by HI tests, were detected in 6 of 115 (5.2%) water deer (*Hydropotes inermis*) serum samples that were collected from a wildlife rescue center in Chungnam Province, South Korea during 2014-2016 [28]. Although IAV antibody detection in this group of mammals may seem unlikely, water deer are known to live within close proximity to water sources in some regions [45]; this could put this species in close contact with waterfowl. Furthermore, water deer exhibit synanthropic tendencies in some regions, as they are some of the most frequent wildlife to come into close contact with farms and livestock barns in South Korea [46]. Other artiodactyl species that are unlikely synanthropes have shown evidence of IAV exposure [33], while some have not, even while living near wetlands on the Arctic Coastal Plain of Alaska with ample opportunities for close interactions with waterfowl [25].

Generically speaking, antibodies to IAVs have been commonly detected in feral swine (*Sus scrofa*) populations. The majority of these detections have been associated with subtypes that are commonly found in domestic swine. However, during the early 1980s, epidemiological studies from Belgium suggested that avian viruses may have produced infections in domestic swine [47]. In the U.S., researchers recently reported that although U.S. feral swine were predominantly exposed to swine IAVs, they may have also been exposed of avian IAVs on a less frequent basis [30]. Based on a relatively small sample size (*n* = 61), one feral swine in Mexico was assessed to be positive for antibodies reactive with an H5N2 virus that was presumed to be avian in origin [31]. In China, however, a small number of feral swine were reported to have been exposed to typical swine viruses (e.g., H1 and H3), but did not react to select avian viruses (e.g., H5 and H9) [48].

## 3. Virus and Viral RNA Detections

Few studies not associated with IAV outbreaks in commercial poultry have investigated mammals for virus and viral RNA of IAVs, although some have assessed antibodies (see above). Virus detection in mammals associated with natural environments is largely dominated by plateau pika, some of which were sampled near Qinghai Lake, China (Table 2). For example, H5N1 was isolated from tissues of five of 147 plateau pika collected near this lake during 2007 [19]. Soon thereafter, a second virus, which was assessed to be an H9N2 subtype, was isolated from tissue samples of one of 138 plateau pika sampled in Qinghai Province [49]. In addition, eight H7N2 isolates obtained from pika living in the same region were recently reported [50].

Of interest, an H13N6 IAV was reported from biological samples collected from a muskrat (*Ondatra zibethicus*) from the Republic of Buryatia (Russia) during the summer of 2000 [51] while an H4N6 IAV (and possibly other H4 viruses) was reported in this species from Russia during the same general time period [52]. Furthermore, two studies have reported H2N2 IAVs in wild muskrats [53,54]. The latest isolate, A/muskrat/Russia/63/2014 (H2N2), was from a sample collected from a wild muskrat in Western Siberia, Russia [54]. Importantly, additional characterization of this virus suggested that it can replicate in mice and mammalian cell lines in the absence of prior host adaptation [65]. The aquatic tendencies of this rodent likely put it into close contact with or within the same environments as many aquatic bird species. For example, it was suggested that the 2014 H2N2 virus may have directly crossed the interspecies barrier from birds to this mammalian species in or near the lake used by both taxa [65]. While typically semi-aquatic in nature, muskrats could be synanthropic in areas where farms or towns border aquatic habitats.

A study investigating IAVs in urban Norway rats (*Rattus norvegicus*) was recently conducted in Boston, Massachusetts, U.S. Eighteen of 163 rats (11%) were positive for viral RNA based upon RT-PCR analyses targeting the IAV matrix gene [55]. Positive samples were detected from oronasal swabs, paw swabs, and/or lung homogenates [55]. The origin of the IAV(s) associated with these RT-PCR positives samples is unknown. However, the authors suggested the possibility of human- and/or avian-origin viruses due to sample locations and the co-occurrence of the rats with key avian species [55]. Additional IAV PCR detections have been reported for small percentages of sampled black rats (*R. rattus*) and house mice (*Mus musculus*) from multiple areas of Bangladesh [56]. However, it is unclear if the reported IAV PCR positive swab samples are likely of avian-origin. Concurrently, these authors also detected IAV PCR positive swab samples from a small number of a synanthropic insectivores identified as Asian house shrews (*Suncus murinus*) [56]. Notably, this insectivore and the majority of the aforementioned rodents are often considered synanthropes, as they are frequently found in peridomestic settings.

During March 2006, a wild stone marten (*Martes fonia*) was confirmed to have a highly pathogenic (HP) H5N1 infection by multiple methods [57]. Prior to euthanasia, this animal, which was living on the Island of Rügen, Germany, exhibited a variety of clinical signs of disease [57]. As previously reviewed, many other mustelid species and allies have shown evidence of exposure to or replication of a variety of IAVs [5]. Although primarily associated with farmed animals, a good example can be found in mink. During the 1980s, an outbreak of an avian-origin IAV was reported in captive mink in Sweden [66]. Furthermore, HP IAVs A/mink/Eastern China/G/2015(H5N1) and A/mink/Eastern China/XB/2015(H5N1) have been isolated from farmed mink in China [67]. Viral detections in mink are not limited to farmed animals, as an additional HP IAV, A/Sweden/mink/2006/V907(H5N1), was isolated from a wild mink in Sweden in a location where wild bird IAV activity had been detected [58,59]. Notably, a recent paper suggested that semi-aquatic mammals, such as mink, could be an important intermediate host in the interspecies spread of avian IAVs to diverse taxa [68]. It should also be noted that an additional mustelid, the domestic ferret (*Mustela putorius furo*), is likely the most widely used animal model for studying IAVs. Due to various similarities ferrets share with humans, this species is used to assess the pandemic potential of certain IAVs [69]. While this review did not discover any naturally occurring or experimental exposures in several additional species of wild mustelids, some of these species, such as various weasels and polecats, are likely synanthropic in some settings. As such, many of these species warrant additional surveillance and experimental studies.

In June 2005, three Owston’s civets (*Chrotogale owstoni*) co-housed in a single enclosure died rapidly of a viral infection; H5N1 IAV was isolated from all three civets [60]. The animals were associated with the Owston’s Civet Conservation Program in Vietnam and were not fed dead poultry, thereby suggesting an unidentified source of infections [60]. Although these were not wild animals and their rarity suggests that they would be uncommon vectors to poultry farms, they are included in this review because a similar, but yet to be described, infection source could be apparent in natural situations and their infections may be representative of more common species in the suborder Feliformia, such as various mongoose species (e.g., *Herpestes* spp.).

Multiple raccoon dogs died from complications with respiratory disease and/or diarrhea in early 2005 in Shandong Province, China [61]. Two of the animals, which were fed chicken carcasses, were confirmed to have an H5N1 IAV infection from assays of lung tissue samples [61]. Similar to the Owston’s civets mentioned above, these animals were not found in wild settings. However, they are included in this review because a similar transmission scenario could readily occur in nature. Further, raccoon dogs can be found in live animal markets in some regions [70].

While IAV antibody detections have been commonly reported in raccoons, few studies have investigated virus and/or viral RNA in this mesocarnivore. However, a recent study reported IAV genetic material in juvenile feral raccoon samples collected in Japan [62]. This finding represents an obvious useful line of future research.

Although not avian-origin, striped skunks (*Mephitis mephitis*) have exhibited tissues positive for the pandemic (H1N1) 2009 IAV [63]. More recently, a possible reverse zoonosis was suspected in striped skunks shown to be infected with A(H1N1)pdm09 clade 6B.1 [64]. Although the source of this virus was likely humans, these natural exposures, coupled with the successful experimental infections of striped skunks with viruses of avian-origin suggests that skunks are possibly naturally exposed to avian viruses when conditions are conducive for transmission [5,71]. However, very little work has attempted to address this question in striped skunks in natural settings.

An IAV survey of feral and domesticated swine was recently reported from Mexico. While multiple swine were assessed to be positive for viral RNA from IAVs of various origins, a single feral pig was positive for a virus described as Feral Swine/Campeche/DMZD-DEFSAL-UIFMVZ19-12 (H5N2) [31]. Although phylogenetic analyses indicated that this virus was in the same clade as both avian and swine viruses, the authors suggested that the virus they sequenced was avian-origin [31].

## 4. Experimental Infections

The bulk of experimental infections of IAVs in synanthropic mammals have involved mesocarnivores, lagomorphs, and rodents (Table 3). Although the methods for assessing the viral titers of these species have varied (Table 3), these data are useful for assessing key species that might be relevant at the wildlife-livestock interface. In most instances, nasal and/or oral viral shedding is reported in the literature, which represent secretions that could lead to transmission in some instances. However, only titers associated with select tissue samples are reported in some papers. Although this is representative of virus replication, it is unclear in these instances if these animals have the capacity to shed the virus in question into the environment in which they live through virus-laden secretions at levels adequate to initiate transmission. However, even if these animals do not shed virus by conventional means (e.g., nasal and oral secretions), they could still pose a transmission risk through predator-prey transmission if the virus is being replicated at sufficient levels in key tissues.

Plateau pika were successfully infected with HP H5N1 in an experimental study [72]. Although assessment of the viral titers shed was not among the objectives of the study, serious lung damage was noted for individuals infected with this virus as compared to the two other subtypes (H1N1 and H3N2) evaluated [72]. A second plateau pika experimental study, which assessed the replication of H9N2 in this species, reported a maximum titer of 10^4.75^ EID_50_/mL from nasal turbinate samples [7]. An additional member of the mammalian order Lagomorpha, the cottontail rabbit (*Sylvilagus* sp.), has been evaluated experimentally for viral shedding (Table 3). Overall, cottontail rabbits have yielded some of the highest peak titers (e.g., 10^7.2^ EID_50_ equivalents/mL) of tested wild mammal species [73]. Of significance, cottontail rabbits were recently shown to be susceptible to IAV infections from very low doses [73], well below the viral doses that have been commonly observed from experimentally infected mallard contamination of small waterbodies [74,75]. To our knowledge, cottontails have successfully shed every strain (n = 5) that they have been inoculated with (both HP and low pathogenic [LP] viruses; Table 3).

Considering the vast number of rodent species found world-wide, a relatively small number of wild rodent species have been experimentally assessed for their abilities to replicate IAVs. House mice (*Mus musculus*) were inoculated with five LP viruses, two of which were isolated from poultry and the other three from wild birds [4]. The highest titer was observed in an H4N6 (wild bird-derived) infected house mouse lung sample collected on 1 DPI (10^6.15^ EID_50_ equivalents/mL) [4]. To date, studies of the Old World bank vole (*Myodes glareolus*) have reported some of the highest potential titers shed (10^6.6^) from a wild rodent animal model [78]. Thirteen-lined ground squirrels *(Ictidomys tridecemilneatus*) successfully replicated a LP H3N8 IAV, but titers associated with viral shedding were not reported [79]. Commensal rats of the genus *Rattus* have also been evaluated for IAV replication. Black rats (*R*. *rattus*) were evaluated for their ability to replicate HP and LP H5 and H7 viruses. Of the seven viruses evaluated, replication was only observed in rats infected with A/whooper swan/Hokkaido/4/2011 (H5N1) with a maximum titer of 10^3.0^ PFU/g of kidney and intestine [81]. In a recently published study, Norway rats (*R*. *norvegicus*) were tested for their ability to replicate four LP viruses derived from poultry and wild birds. Viral replication was highest in rats inoculated with the wild bird viruses, with peak titers of 10^4.78^ EID_50_ equivalents/mL from a nasal turbinate sample of a rat inoculated with an H4N6 virus and 10^5.45^ EID_50_ equivalents/mL from a caudal lung sample of a rat inoculated with an H3N8 IAV [80]. In artificial barnyard settings with rats and multiple avian species present, contact rats exposed to the same environment as infected mallards showed no evidence of viral shedding by the oral route but did show evidence of seroconversion for one of the two LP viruses tested; this study did not evaluate organs [74]. The relatively high titers detected in organs of some small rodent species suggests that IAV transmission could possibly occur through the ingestion of these small mammals, but this will require experimentation to determine if this is the case. Furthermore, the probability of this type of predator-prey transmission may be more likely when rodents infected with HP IAVs are ingested.

A limited number of experimental infections and transmission studies have been conducted on raccoons. In a study that evaluated multiple ecological routes of IAV transmission to raccoons, only a relatively high dose of water successfully transmitted the virus to this species, producing a maximum titer of 10^4.2^ EID_50_ equivalents/mL in nasal swab samples following ingestion of LP H4N6 (wild-bird origin) virus-laden water [82]. A second raccoon study, which evaluated the viral shedding following nasal inoculation with an emergent Asian H7N9 IAV, produced a maximum titer of 10^5.2^ pfu/mL from nasal flushes [76]. A third study, which evaluated IAV shedding of raccoons experimentally infected with an H4N8 poultry-derived virus, reported very low levels of shedding, with a maximum titer of 10^1.1^ EID_50_ equivalents/mL [22].

Thus far, striped skunks have shed some of the highest titers of the peridomestic mesocarnivores tested to date, producing nasal shedding levels of 10^6.0^ to 10^6.4^ for two LP strains of IAV [5,76]. Of importance, striped skunks can produce nasal shedding for extended periods. Relatively high titers to some IAV subtypes have also been noted in experimentally infected mink [40,85]. For example, of the six viruses tested, nasal swab titers were highest from a mink experimentally inoculated with an avian H3N8 IAV, peaking at ≥10^6.0^ EID^50^/swab [85]. A second study, in which titers of nasal swabs were not performed, reported very high titers from select tissue samples, up to 10^7.2^ EID_50_/g of turbinate samples from a mink infected with an H7N2 virus of avian-origin [40]. However, this mink was inoculated with 10^8.7^ EID^50^ [40], a dose that is orders of magnitude higher than has been used by many other experimental studies.

Based upon the finding of presumably avian IAV antibodies in feral swine, an experimental challenge was conducted on this species using two avian viruses [30]. Swine inoculated with a mallard-derived H3N2 virus developed a maximum titer of 10^2.5^ EID_50_/mL from nasal wash samples and up to 10^3.5^ EID_50_/g from lung tissue [30]. Titers were not evaluated and/or reported for the second virus that was tested.

## 5. Potential Mammalian Involvement in Outbreaks of IAV on Poultry Farms

The potential role of wild mammals in IAV epidemiology at poultry farms has elicited conflicting opinions. While one questionnaire-based study indicated that mammalian wildlife on farms represented a significant risk factor for LP IAV infection [9], a more recent survey noted a very marginal association of the presence of small mammals near poultry barns with reduced odds of HP infection at case farms [87]. However, the authors of the latter study acknowledged the potential involvement of mammalian trafficking of IAVs farm-to-farm and indicated that wild mammal involvement in IAV epidemiology should receive further evaluation [87].

Three recent investigations assessed potential wildlife involvement in H5 IAV case clusters in commercial poultry facilities in the Midwestern U.S. during and/or following the 2015 outbreak of clade 2.3.4.4 IAVs. The first sampled 190 individual mammals (primarily house mice, *Mus musculus*) across five positive farms in northwestern Iowa. Although a large sample size was obtained for multiple sample types (e.g., oral swabs, nasal washes, serum, and tissues), none provided evidence of viral RNA or antibodies reactive with IAV [1]. A second study evaluated potential wildlife involvement in a previously HP H5 infected commercial poultry farm several months after poultry depopulation. Of the 47 swab samples collected from mammals captured on a previously IAV affected farm, none were positive for viral RNA; however, a single *Peromyscus* sp. mouse was assessed to be antibody positive by ELISA but was negative by HI tests [88]. A third study evaluated small mammals associated with Iowa poultry farms, some of which were located in counties that had HP IAV activity during 2015, several months after the 2015 outbreak had ceased [89]. None of the samples (a relatively small number of external and internal swabs) tested positive by PCR, nor did any of the subset of blood samples collected from small mammals (house mice, *Peromyscus* sp., meadow voles [*Microtus pennsylvanicus*], Norway rats, northern short-tailed shrews [*Blarina brevicauda*]) test positive for IAV antibodies [89]. All of these studies typically sampled mammalian wildlife weeks to months following depopulation of their respective farms. Thus, the lack of positive viral RNA samples in the three studies may be confounded by the timing of the sampling [1,88], as these mammals that are potential spillover hosts would most likely be exposed to IAVs during active poultry outbreaks or during epizootics in wild birds. As such, future epidemiological investigations should endeavor to sample wildlife during poultry outbreaks as near to real time as logistically possible if virus or viral RNA is targeted.

A scant number of more historical epidemiological investigations have been conducted in areas near affected poultry premises in the northeastern U.S. during the 1980s and 1990s. One investigation evaluated lung tissue from 245 house mice and 24 Norway rats in a Pennsylvania quarantine zone associated with an H5N2 IAV in commercial poultry and found no evidence of infection by virus isolation in these species [90]. In addition, none of the serum samples from 103 house mice nor any of the 22 serum samples from Norway rats yielded evidence of antibodies against H5 or N2 by HI tests [90]. A second study, which evaluated tissue samples from a fraction of 141 house mice collected from 10 poultry facilities in Pennsylvania for IAV exposure, did not produce any viral isolates [91].

Although it was not conducted during an IAV outbreak, a recent study identified several wild mammal species utilizing areas associated with a commercial free-range layer farm in the Netherlands [92]. For example, red fox (*Vulpes vulpes*), stone marten (*Martes foina*), hares and rabbits (*Lepus europaeus* and *Oryctolagus cuniculus*), and various rodent species were documented using different parts of the aforementioned poultry facility [92]. Notably, select members from the mammalian families associated with these species have shown IAV replication ability or have been assessed to be naturally exposed to some IAVs (Table 1, Table 2 and Table 3).

## 6. Conclusions and Future Directions

**Figure 2:**
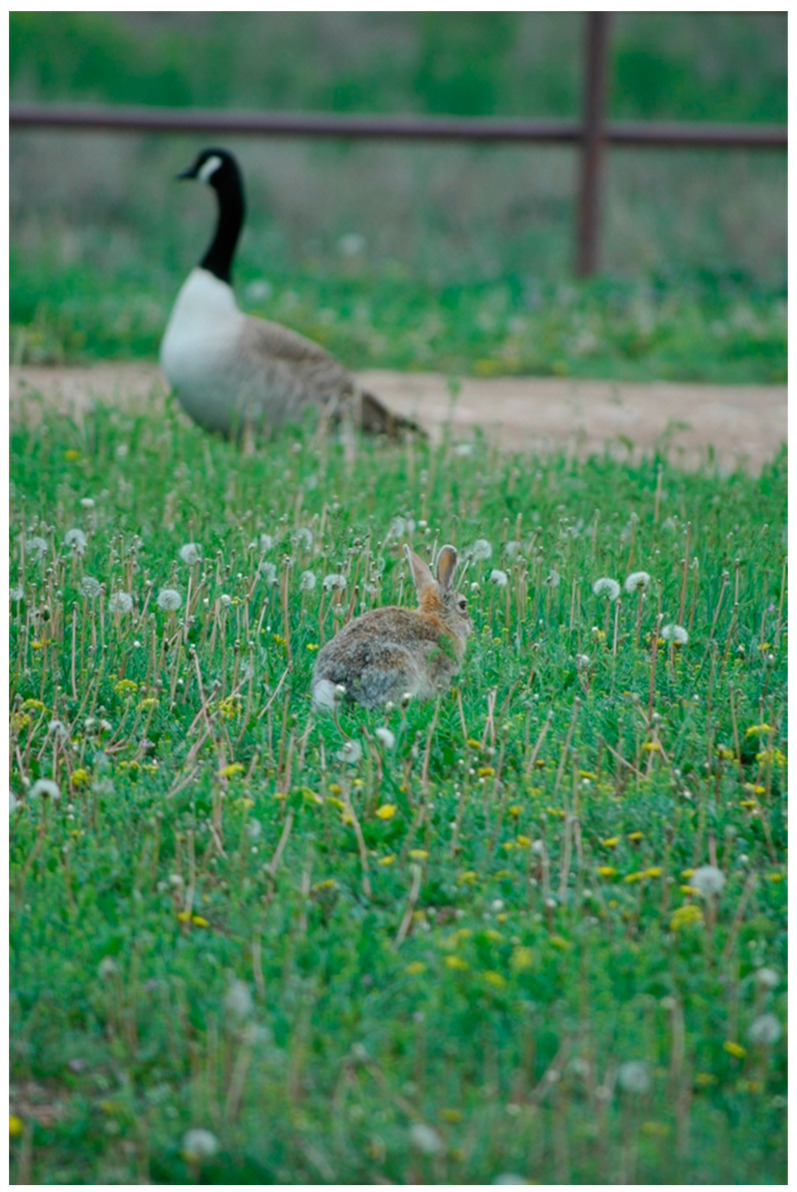
A cottontail rabbit (*Sylvilagus* sp.; foreground) and a Canada goose (*Branta canadensis*; background) foraging in close proximity to each other, a scenario in which interspecific transmission of various pathogens, including influenza A viruses, could occur. A similar transmission mechanism associated with shared foraging sites has been proposed for plateau pika (*Ochotona curzoniae)* and waterfowl (e.g., bar-headed geese [*Anser indicus*] and ruddy shelducks [*Tadorna ferruginea*]) at Qinghai Lake, China [19].

**Figure 3:**
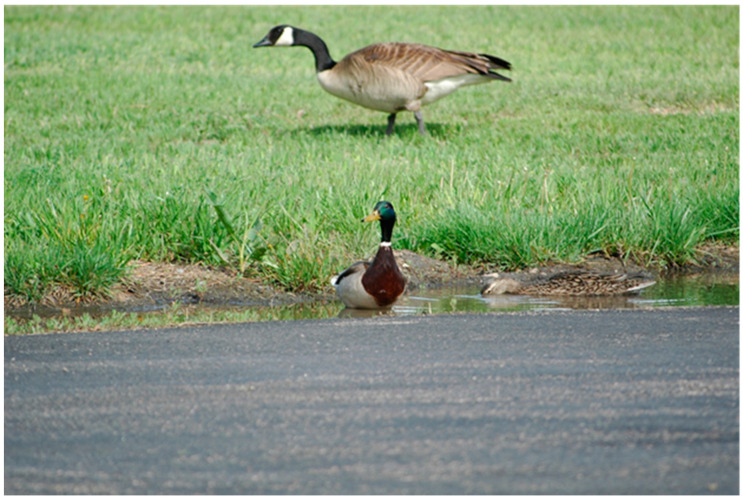
Mallards (*Anas platyrhynchos*; foreground) and a Canada goose (*Branta canadensis*; background) utilizing the same small water source. This photograph represents a situation in which influenza A virus transmission could occur from a dabbling duck to an avian species that primarily forages in terrestrial habitats, occasionally in close proximity to mammals (see Figure 2).

The behavioral tendencies of certain mammalian synanthropes could facilitate their acquisition of IAVs. Raccoons, for example, often forage in aquatic environments, which could put them in contact with bird carcasses, bird feces, or virus-laden water, all of which have been postulated as potential transmission mechanisms to this species [22,82]. Although successful transmission from virus-laden avian carcasses to red foxes was noted for a HP IAV [83], avian carcasses infected with a LP IAV failed to produce transmission to raccoons [82]. However, virus-contaminated water at relatively high titers did produce viral replication and seroconversions in raccoons [82]. Cottontail rabbits, as another example, typically forage in grassy areas, where they may utilize the same feeding areas of certain waterfowl species, especially geese (Figure 2). Considering that shared foraging sites with waterfowl was a suspected transmission mechanism of avian IAVs to plateau pika [19], a similar transmission mechanism may be possible for cottontail rabbits, especially considering that they are susceptible to low viral doses by multiple routes [73]. While the aforementioned system is associated with terrestrial foraging sites, small water bodies, especially those that attract multiple wildlife species (Figure 3), could have interspecific transmission implications for both birds and mammals.

In addition to their ability to replicate and shed various IAVs, species-specific ecological characteristics should also be taken into account when evaluating the likelihood of a mammalian species to be involved in IAV epidemiology. For example, diet (e.g., commonly feeding upon birds) and gregariousness [17], home range sizes, population densities, and geographical distributions (e.g., small or wide-scale) could all be important facets in intra- and/or interspecific transmission of IAVs.

We have observed spilled grain associated with feed hoppers at multiple commercial poultry facilities. The frequency at which this occurs is largely based upon the age of a facility, as well as the maintenance schedule of the facility. This type of unnatural food subsidy can act as an attractant to wildlife [1]. Thus, in this scenario, the movement of an IAV into a poultry facility could involve multiple steps. For example, a highly mobile raccoon or striped skunk, either of which can successfully replicate IAVs [5,22,82], could acquire an IAV infection while foraging in a natural area or wetland [22] (Figure 1). Considering that both of these mesocarnivores have been shown to shed some subtypes for multiple days [5,76], the animal could forage on the spilled grain adjacent to a poultry barn following its IAV exposure, potentially depositing virus-laden secretions. Subsequently, a rodent, such as a house mouse, could move the virus inside a poultry facility through permeable features of the barn (Figure 1). Alternatively, during periods when active IAV infections are occurring in poultry facilities, a rodent could potentially facilitate viral spread by acquiring a virus in a poultry house and subsequently be scavenged or predated upon outside of the facility [4]. Of significance, a mammal does not necessarily have to enter a poultry barn to facilitate transmission, as an IAV brought to locations near a poultry facility could be transported into a facility on footwear or equipment in instances when routine or effective decontamination procedures are not employed.

In most instances where HP viruses are not circulating within a poultry facility, a mammalian synanthrope may be more likely to bring a LP H5 or H7 to areas near a poultry facility as compared to a HP virus of these subtypes. One example can be found in raccoons. Considering that raccoons commonly exploit a variety of natural and anthropogenically modified habitats, an individual that acquired an IAV infection from scavenging a diseased bird [22] or through environmental contamination [82] could readily transport a virus to areas near poultry operations [22]. Similar to rodents [11], this could potentially be accomplished through virus replication or through mechanical transmission. Although initially low pathogenic, a LP H5 or H7 virus can mutate to a highly pathogenic form when introduced into commercial poultry flocks [93].

**Figure 4:**
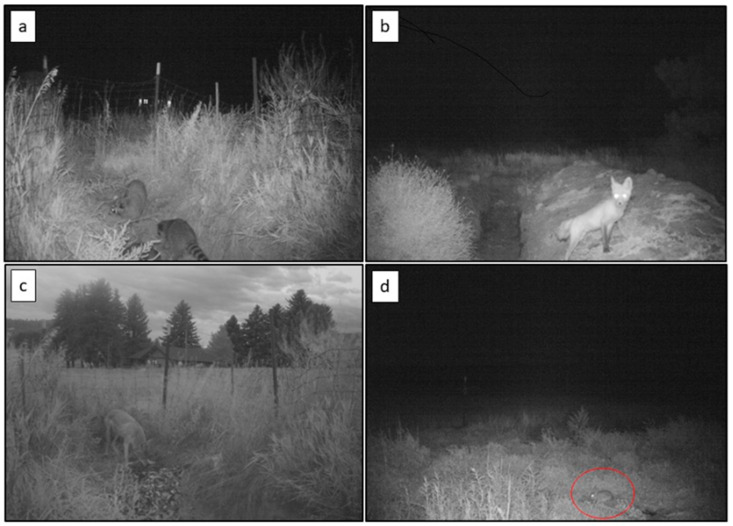
(**a**) Raccoons (*Procyon lotor*), (**b**) a red fox (*Vulpes vulpes*), (**c**) and a coyote (*Canis latrans*) scavenging in vertebrate mortality pits and (**d**) a cottontail rabbit (*Sylvilagus* sp.) foraging immediately adjacent to a mortality pit. These feeding events could result in pathogen transmission under certain conditions [94].

Alternative to acquiring an IAV from wild waterfowl associated with natural areas, a synanthropic mammal, even those of sizes too large to readily enter poultry barns, could acquire an IAV infection if IAV was present in the poultry facility under certain conditions. For example, poultry facilities use a variety of disposal methods (e.g., composting, incineration, rendering, mortality pits, etc., which are often dependent on the size of the facility) for poultry carcasses accumulated from normal losses during production activities. In some facilities, composting is conducted in locations that wild animals could readily access. Furthermore, carcasses intended for rendering are often stored in containers on farms; these containers may or may not be secure from wildlife. In addition, mortality pits are occasionally used to dispose of animal carcasses (e.g., dead fish and wildlife, etc.; Figure 4). However, the animals disposed of in these pits are not always immediately buried. Thus, considering that wildlife can be naturally attracted to a bird carcass (potentially infected with IAV in some instances) [88,94], many of these scenarios could represent transmission routes back to mammalian wildlife when active IAV infections are occurring on a poultry facility.

The natural behaviors of various mammal species could modulate the risks that they pose to poultry operations. Wild rabbits on farms, as one example, are likely recurrent residents of the facilities, as plentiful sustenance is likely present for this small herbivore in most instances. More mobile mesocarnivores, such as raccoons, striped skunks, and Virginia opossums (*Didelphis virginiana*), could occasionally visit farmsteads at night during nocturnal foraging excursions. Thus, from a behavioral context, a rabbit could acquire an influenza infection through shared foraging sites with waterfowl such as geese. This transmission scenario has been postulated for an Old World lagomorph species [19]. Raccoons and other mesocarnivores, on the other hand, could visit a wetland area potentially contaminated with IAV and subsequently visit one or more poultry facilities during the same night [22].

Although a strong epidemiological link of a mammal trafficking IAV to a commercial poultry facility has yet to be demonstrated, a recent study clearly demonstrated the ability of select mammal species to transmit IAV back to an avian reservoir species through shared resources [71]. In addition, mechanical transmission and viral replication of rodents have been suggested as potential pathways that IAV can infiltrate commercial poultry, even in farms with biosecurity measures in place [11]. The numerous papers suggesting that mammals can successfully replicate avian IAVs, confounded with the lack of evidence of mammalian exposures on poultry farms, exemplifies the need to conduct on-farm epidemiological investigations as quickly as possible, preferably during an active IAV outbreak in poultry and before the affected poultry have been depopulated. Furthermore, epidemiological investigations at these premises would shed additional light as to the role of wildlife in IAV ecology at the farm level. In addition, known “hot spots” of IAV in wild birds [95] associated with habitats that also support key mammalian species, should be further evaluated elsewhere for IAV exposure in wild mammals [96].

## Figures and Tables

**Table 1:** Select antibody detections of influenza A viruses from various wild synanthropic mammalian species in natural and semi-natural settings and similar species in captive settings (e.g., farmed wildlife). Certain species that may only occasionally exhibit these synanthropic tendencies are included.

Common Name ^a^	Scientific Name	Location	Citation
Plateau pika	*Ochotona curzoniae*	China	[7,19]
Daurian pika	*O. dauurica*	Russia	[16]
“Rats”	Presumably *Rattus* sp.	Hong Kong	[20]
“Sewage rats”	Presumably *Rattus* sp.	Egypt	[21]
House mouse	*Mus musculus*	U.S.	[4]
Transbaikal hamster ^b^	*Cricetulus pseudogriseus*	Russia	[16]
Campbell’s dwarf hamster ^b^	*Phodopus campbelli*	Russia	[16]
Brandt’s vole	*Lasiopodomys brandtii*	Russia	[16]
Mongolian gerbil	*Meriones unguiculatus*	Russia	[16]
Daurian souslik	*Spermophilus dauricus*	Russia	[16]
Raccoon	*Procyon lotor*	U.S.	[22]
		Japan	[23,24]
Arctic fox	*Vulpes lagopus*	U.S.	[25]
“Fox” ^c^	Not listed	China	[26]
Black-backed jackal	*Canis mesomelas*	Namibia	[17]
Raccoon dog ^c^	*Nyctereutes procyonoides*	China	[26]
Far-eastern wild cat	*Prionailurus bengalensis euptilurus*	Russia	[27]
Leopard cat	*Prionailurus bengalensis*	Korea	[28]
American mink	*Neovison vison*	Spain	[38]
Water deer	*Hydropotes inermis*	Korea	[28]
Feral swine	*Sus scrofa*	U.S.	[30]
		Mexico	[31]

^a^ This table is not intended to be an exhaustive list of all wild mammal exposures to influenza A viruses (IAV), as the focus of this paper is mammalian synanthropes. Some additional non-synanthropic species are listed in the text to bring attention to IAV exposures in select taxa. ^b^ Results presented generically as “hamster” in original article. ^c^ Animals were farmed but are listed to show evidence of exposure in this species. Animals were likely fed uncooked poultry products.

**Table 2:** Select virus and nucleic acid detections of influenza A viruses from synanthropic mammalian species in natural and semi-natural settings.

Common Name	Scientific Name	Type	Subtype	Location	Citation
Plateau pika	*Ochotona curzoniae*	Virus	H5N1	China	[19]
		Virus	H9N2	China	[49]
		Virus	H7N2	China	[50]
Muskrat	*Ondatra zibethicus*	Virus	H13N6	Russia	[51]
		Virus	H4N6	Russia	[52]
		Virus	H2N2	Russia	[53,54]
Norway rat	*Rattus norvegicus*	RNA ^a^	ND ^b^	U.S.	[55]
Black rat	*Rattus rattus*	RNA	ND ^b^	Bangladesh	[56]
House mouse	*Mus musculus*	RNA	ND ^b^	Bangladesh	[56]
Asian house shrew	*Suncus murinus*	RNA	ND ^b^	Bangladesh	[56]
Stone marten	*Martes fonia*	RNA/Virus	H5N1	Germany	[57]
“Mink”	*Neovison vison* ^c^	Virus	H5N1	Sweden	[58,59]
“Mink”	Not reported ^d^	Virus	H9N2	China	[26]
Owston’s civet	*Chrotogale owstoni*	Virus	H5N1	Vietnam	[60]
Raccoon dog	*Nyctereutes procyonoides*	Virus	H5N1	China	[61]
Raccoon	*Procyon lotor*	RNA	ND ^b^	Japan	[62]
Striped skunk	*Mephitis mephitis*	RNA/Virus	H1N1 ^e^	Canada	[63,64]
Feral swine	*Sus scrofa*	RNA	H5N2	Mexico	[31]

^a^ Authors suggested origin of viral RNA could be human or avian based upon capture locations of sampled animals. ^b^ ND = not determined or not reported. ^c^ Presumably an American mink (*Neovison vison*; reported in reference [58] as *Mustela vison*); the American mink was introduced to Sweden during the early 1900s. Reference [59] did not report a scientific name. ^d^ Mink species not listed. Virus was isolated from a farmed mink fed uncooked poultry products. ^e^ A(H1N1) pdm09. Virus is not avian-origin but is included for evidence of natural IAV infections in striped skunks. Virus was isolated in second study referenced.

**Table 3:** Viral shedding of influenza A viruses (generally avian or avian-origin but isolated from an alternative host [e.g., Anhui]) by select experimentally infected mammalian synanthropes.

Common Name	Scientific Name	Subtype	Pathotype ^b^*	Exposure Method	Maximum Titer ^f^*	Citation
Plateau pika	*Ochotona curzoniae*	H5N1 ^a^	HP	IN ^c^*	ND	[72]
		H9N2 ^b^	LP	IN	4.8 ^h^*	[7]
Cottontail rabbit	*Sylvilagus* sp.	H4N6 ^c^	LP	IN	7.2	[73]
		H7N9 ^d^	LP	IN	5.8	[76]
		H5N8 ^e^	HP	IN	3.5	[77]
		H5N2 ^f^	HP	IN	3.9	[77]
		H5N2 ^g^	HP	IN	5.3	[77]
House mouse	*Mus musculus*	H3N6 ^h^	LP	IN	2.4 ^i^*	[4]
		H3N8 ^i^	LP	IN	4.5 ^i^*	[4]
		H4N6 ^j^	LP	IN	4.3 ^i^*	[4]
		H4N8 ^k^	LP	IN	2.1 ^i^*	[4]
		H6N2 ^l^	LP	IN	2.1 ^i^*	[4]
Bank vole	*Myodes glareolus*	H7N1 ^m^	HP	IN	4.9	[78]
		H5N1 ^n^	HP	IN	6.6	[78]
Thirteen-lined ground squirrel	*Ictidomys tridecemlineatus*^a^*	H3N8 ^o^	LP	IN	ND ^g^*	[79]
Norway rat	*Rattus norvegicus*	H6N2 ^l^	LP	IN	3.5 ^i^*	[80]
		H4N8 ^k^	LP	IN	1.7 ^i^*	[80]
		H4N6 ^j^	LP	IN	4.8 ^i^*	[80]
		H3N8 ^i^	LP	IN	5.5 ^i^*	[80]
Black rat	*Rattus rattus*	H5N1 ^p^	HP	IN	3.0 ^j^*	[81]
Raccoon	*Procyon lotor*	H4N6 ^c^	LP	Virus-laden water	4.2	[82]
		H7N9 ^d^	LP	IN	5.2	[76]
		H4N8 ^k^	LP	IN	1.1	[22]
Striped skunk	*Mephitis*	H4N6 ^c^	LP	IN	6.0	[5]
		H7N9 ^d^	LP	IN	6.4	[76]
		H1N1 ^q^	LP	ICE ^d^*/IN	ND	[79]
		H3N8 ^o^	LP	ICE/IN	ND	[79]
Red fox	*Vulpes vulpes*	H5N1 ^r^	HP	IT or BC ^e^*	5.2	[83]
“Fox”	Not listed	H9N2 ^s^	LP	IN or Contact	Negative	[26]
Mink	*Neovison vison*	H9N2 ^t^	LP	IN	ND	[41]
		H7N2 ^u^	LP	IN or Contact	7.2 ^k^*	[40,84]
		H4N1 ^v^	LP	IN	4.5 ^k^*	[40]
		H3N8 ^w^	LP	IN	6.3	[85]
		H11N4 ^x^	LP	IN	5.3	[85]
		H7N7 ^y^	Not reported	IN	5.5	[85]
		H8N4 ^z^	LP	IN	6.0	[85]
		H5N3 ^aa^	Not reported	IN	4.5	[85]
		H9N2 ^bb^	LP	IN	4.3	[85]
“Mink”	Not listed	H9N2 ^s^	LP	IN or Contact	4.2	[26]
“Mink”	Not listed	H9N2 ^cc^	LP	IN	4.5	[86]
Raccoon dog	*Nyctereutes procyonoides*	H9N2 ^s^	LP	IN or Contact	Negative	[26]
Feral swine	*Sus Scrofa*	H3N2 ^dd^	LP	IN	2.5	[30]
		H6N2 ^ee^	LP	IN	ND	[30]

^a^ A/greatblack-headedgull/Qinghai/1/2009(H5N1); ^b^ A/Chicken/Shandong/Li-1/2009(H9N2); ^c^ A/Mallard/CO/P70F1-03/08(H4N6); ^d^ A/Anhui/1/2013(H7N9)[humanisolateofavianorigin]; ^e^ A/gyrfalcon/Washington/41088-6/2014(H5N8); ^f^ A/Northern pintail/Washington/40964/2014(H5N2); ^g^ A/turkey/Minnesota/9845-4/2015(H5N2); ^h^ A/Mallard/OR/A00047710/06(H3N6); ^I^ A/Wild bird/CA/187718-36/08(H3N8); ^j^ A/mallard/CO/P66F1-5/08(H4N6); ^k^ A/CK/AL/75(H4N8); ^l^ A/CK/CA/S0408793/04(H6N2); ^m^ A/ostrich/Italy/2332/2000(Os/H7N1); ^n^ A/turkey/Turkey/1/2005(Tk/H5N1); ^o^ A/DK/1518/81(H3N8); ^p^ A/whooper swan/Hokkaido/4/2011(H5N1); ^q^ A/Goose/Mn-1661/81(H1N1); ^r^ A/whooper swan/Germany/R65-1/2006(H5N1); ^s^ A/Mink/Shandong/F10/2013(H9N2) [virus was isolated from a farmed mink fed uncooked poultry products]; ^t^ A/Chicken/Hebei/4/2008(H9N2); ^u^ A/duck/Hokkaido/5/77(H7N2); ^v^ A/budgerigar/Hokkaido/1/77(H4N1); ^w^ A/duck/Hokkaido/8/80(H3N8); ^x^ A/duck/Miyagi/47/77(H11N4); ^y^ A/swan/Tottori/42/80(H7N7); ^z^ A/duck/Miyagi/95/77(H8N4); ^aa^ A/duck/Miyagi/54/76(H5N3); ^bb^ A/duck/Niigata/747/79(H9N2); ^cc^ A/Mink/Shandong/F6/2013(H9N2) [virus was isolated from a farmed mink that were likely fed uncooked poultry products]; ^dd^ A/mallard/Wisconsin/A00661712/2009(H3N2); ^ee^ A/mallard/Ohio/648/2002(H6N2); ^a^* Listed in original paper as *Spermophilus tridecemlineatus*; ^b^* Pathotype as assessed in poultry; ^c^* IN = intranasal; ^d^* ICE = animals fed infected chicken embryos; ^e^* IT = Intratracheal, BC = Infected bird carcasses, maximum titer was associated with IT infection; ^f^* Presented as log_10_ “units”/mL. Some studies used quantitative PCR techniques (e.g., PCR EID_50_ equivalents/mL, Copies/µL [converted to mL]), and others used virus isolation techniques. Data associated with tissues is included in some instances; ^g^* ND = not done or not reported; ^h^* Maximum titer was assessed from nasal turbinates; ^i^* Highest titers reported were associated with nasal turbinate or lung samples; ^j^* Titer was associated with intestine and kidney samples; ^k^* Highest titer was from a nasal turbinate sample. Titers analysis of nasal swabs was not conducted.
